# Extraskeletal Ewing’s sarcoma of the mediastinum: Case report

**DOI:** 10.3389/fonc.2023.1074378

**Published:** 2023-01-26

**Authors:** Aldo Caltavituro, Roberto Buonaiuto, Fabio Salomone, Rocco Morra, Erica Pietroluongo, Pietro De Placido, Marianna Tortora, Annarita Peddio, Fernanda Picozzi, Margaret Ottaviano, Mirella Marino, Sabino De Placido, Giovannella Palmieri, Mario Giuliano

**Affiliations:** ^1^ CRCTR Coordinating Rare Tumors Reference Center of Campania Region, Naples, Italy; ^2^ Rare Tumors Coordinating Center of Campania Region (CRCTR) Coordinating Rare Tumors Reference Center of Campania Region, Naples, Italy; ^3^ Division of Medical Oncology, Azienda Ospedaliera di Rilievo Nazionale (A.O.R.N.) dei COLLI “Ospedali Monaldi-Cotugno-Centro Traumatologico Ortopedico (CTO)”, Naples, Italy; ^4^ Unit of Melanoma, Cancer Immunotherapy and Development Therapeutics, Istituto Nazionale Tumori IRCCS Fondazione Pascale, Napoli, Italy; ^5^ Department of Pathology, Regina Elena National Cancer Institute, Rome, Italy

**Keywords:** case report, Ewing sarcoma, multidisciplinary management, thoracic oncology, misdiagnosis cancer

## Abstract

**Background:**

Ewing sarcoma (ES) represents the second most common malignant bone tumor in children and young adults. ES is not a frequent finding in sites different from the skeletal. Common sites of appearance of ES are lower extremities, the pelvis, paravertebral spaces and head and neck. Primary extraskeletal ES located in the anterior mediastinum are very rare. These neoplasms should be discussed in specialized contests with a high volume of patients treated. Here, we present an uncommon mediastinal mass challenging in its characterization and management.

**Case description:**

A thirty-year-old woman performed a thoracic CT scan for dyspnea and persistent cough. Imaging showed a solid mass of 14 x 11 cm involving the left thorax with mediastinal deviation to the right side. Patient underwent an en bloc resection of the mass. Initial histological examination was suggestive for B3 thymoma/thymic carcinoma. Patient was then referred to our rare tumor reference center where a histological review excluded the diagnosis of thymic/thymoma neoplasms meanwhile a third revision assessed a diagnosis of ES. Patient refused adjuvant chemotherapy due to her desire of maternity and radiation therapy was not indicated because surgery was performed too many months earlier. A close follow-up was considered. After a few months the patient relapsed and first line chemotherapy was proposed. She reached a complete response at the first evaluation maintained also at the end of the protocol. In order to consolidate the obtained response, high dose chemotherapy followed by autologous stem cell transplantation (HDCT/ASCT) was suggested and the patient agreed.

**Conclusions:**

This case underlined that, potentially, ES can arise from any soft tissue site in the body, even in rare sites such as mediastinum. The evaluation of expert centers was critical to establish a correct diagnosis and therapeutic approach in this complex case. Taking into account the time lasting from the diagnosis and the aggressiveness of this kind of neoplasm, frequently relapsing, the patient after a multidisciplinary discussion was a candidate for a multimodal treatment.

## Introduction

Adult soft tissue and visceral sarcomas are rare tumors, with an estimated incidence averaging of 4– 5/100.000/year in Europe that account for less than 1% of all tumors (1). They include over 80 different histological subtypes that differ one from another in terms of incidence, treatment and prognosis (2).

Although they can arise from different sites, extremities and abdomen are most commonly involved (3).

Sarcomas can origin also from the mediastinum even if this location is not the most frequent one (4– 5).

The diagnosis of these types of tumors can be very challenging and a multidisciplinary approach is required in order to figure out the most appropriate management.

A lot of professional figures are involved in this field: pathologists, radiologists, thoracic surgeons, oncologists and many more.

ES is a high-grade round cell sarcoma (RCS) that generally affects bones and soft tissues, particularly in children and young adults. Ewing sarcoma family is a group of neoplasms containing but not limited to ES, also peripheral primitive neuroectodermal tumors belong to this group. Principles applied for bone ES are also extended to primary extraskeletal locations. The discovery of an ES in the mediastinum is not very common. Here we described a case of primary extraskeletal mediastinal ES.

This article aimed to show an uncommon finding of ES of the mediastinum and its complex and multidisciplinary management.

We present the following case in accordance with the CARE reporting checklist.

## Case presentation

A thirty-years old woman, fifteen cigarettes/die smoker, without relevant medical history, referred to the emergency service complaining of persistent cough and unusual dyspnea.

During the physical exam a silent auscultatory area in the left upper hemithorax was found.

A chest X-Ray showed, in the anterior mediastinum, a huge mass (19 cm x 11 cm x 12 cm) with defined margins expanding in the superior and medium left hemithorax consistent with the auscultatory abnormality. The mass induced a contralateral dislocation of mediastinal structures and upper airways compression.

**Figure 1:**
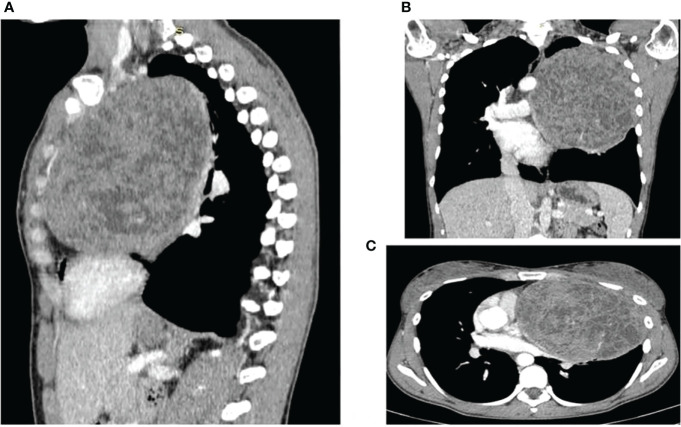
CT scans showing the presence and anatomical relation of the heteroplastic mass. **(A)** sagittal plane **(B)** coronal plane **(C)** transverse plane.

Computed tomography (CT) scan confirmed a solid mass characterized by dishomogeneous contrast enhancement, bordering to the costal pleura on the lateral side and to mediastinal great vessels, which compressed the superior lung lobe (**Figure 1**).

18- Fluorodeoxyglucose-Positron Emission Tomography/Computed Tomography scan (18F-FDG PET/CT scan), performed later, did not reveal any sign of metabolic or morphological suspicion of distant disease.

The patient was a candidate for up-front surgery.

Despite the major surgery, the patient did not experienced remarkable post-operative complications and achieved a complete recovery. In addition, neither functional nor aesthetical impairment occurred.

The lesion was removed en bloc with the thoracic wall and the intercostal muscles; during surgery the anterior arches of the third, fourth and fifth ribs were excised and a segmental resection of the lingula and the anterior segment of the superior lobe of the left lung was performed.

Pathological examination was performed and, macroscopically, the excised lesion appeared as a spongy and gray encapsulated mass (16.5 x 14 x 8 cm) with white subcapsular areas and blood spots; microscopically, small size neoplastic cells with vesicular nucleus, poor cytoplasm and lobular growth were described.

The immunohistochemical analysis revealed a positivity for P63, CD99, CD117 and vimentina with poor lymphatic infiltrations (CD45+) and negativity to: MPO, TdT, CD3, CD4, CD5, CD30, CD34, CD43, CD68, AFP, HGG, Inhibin A, CK AE1/AE3, CK7, CK20, EMA, PLAP, Calretinin, SMA, Desmine, Synaptophysin, S100, P53. Ki-67 proliferative index was 40%.

All these features were suggestive for a well differentiated thymic carcinoma, also known as B3 thymoma, according to the WHO classification (6). AJCC/UICC TNM 8th edition classified this neoplasm as pT1b (Stage 1 Masaoka modified staging) and according to the last guidelines she underwent a close clinical and radiological follow up (7).

**Figure 2:**
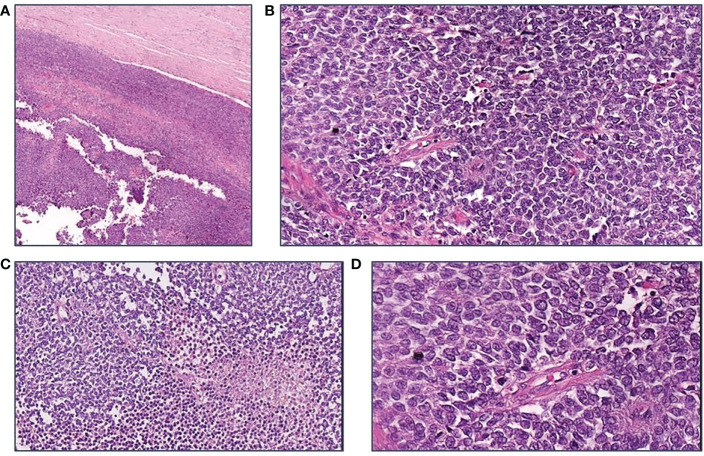
Morphological features of the tumor. **(A)** H$E, 40x: A thick fibrous capsule surrounds the large tumor; **(B)** H$E, 200X; Richly cellular sheets. Cells have scant cytoplasm and vesicular irregular nuclei; frequent mitoses are seen; no lymphocytes are found; **(C)** H$E, 200X: Necrotic foci are scattered; **(D)** H$E, 400X: The cells, rather small, form sheets with a sort of palisading around vessels.

**Figure 3:**
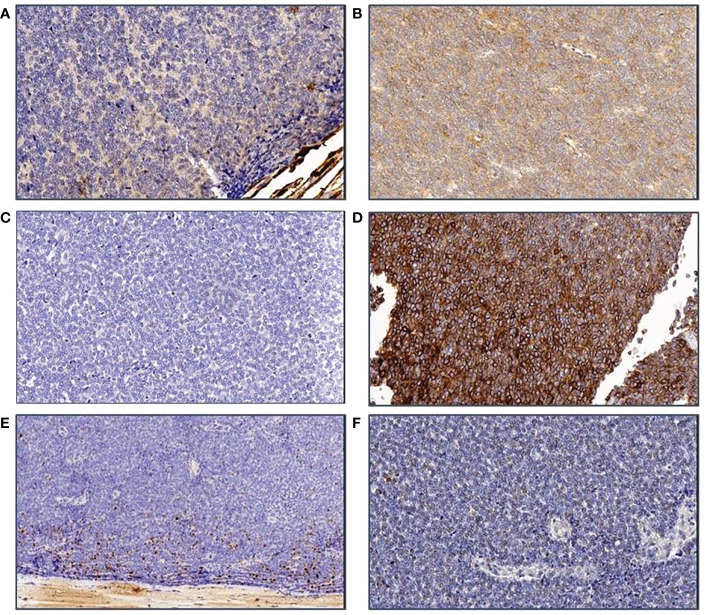
Immunohistochemical features of the tumor. **(A)** ker AE1/3 staining: The tumor cells are negative; **(B)** CD117: A slight unspecific staining is observed; **(C)** ker MNF116 is also neagtive in tumor cells; **(D)** CD99 membrane staining is clearly seen in all cells; **(E)** The Ki-67 staining shows that only the peripheral part of the tumor is marked, probably because of preanalytical damage of tumor tissue; **(F)** Synaptophysin marking is negative in tumor cells.

The patient was referred to the Coordinating Rare Tumors Reference Center of Campania (CRCTR) where an immunochemical and histological revision of the material was requested (**Figures 2**, **3**).

A second opinion from a center with high expertise in thymic malignancies was requested.

The negativity to different types of cytokeratins, the elevated mitotic index, the rare presence of classical thymic markers led to the exclusion of a diagnosis of thymoma or thymic carcinoma. The occasional positivity to P63 was not specific for an epithelial neoplasm, instead the morphological pattern and the only positivity for CD99 were actually consistent with a diagnosis of undifferentiated sarcoma.

Genetic analyses, conducted using next generation sequencing (NGS) technology did not reveal any relevant mutations suitable for diagnostic characterization or therapeutic strategies; in fact no mutations were found in c-KIT gene nor in other candidate genes such as: PDGFR, IDH1, IDH2, PTEN, HRAS, NRAS, GNAQ, BRAF, CTNNB1, TP53, H3F3A, RET, MAP2K1, NF1, GNAS, GNA11.

Furthermore, the transcriptome was analyzed for the following 26 genes: ALK, CAMTA1, CCNB3, CIC, EPC1, EWSR1, FOXO1, FUS, GLI1, HMGA2, JAZF1, MEAF6, MKL2, NCOA2, NTRK3, PDGFB, PLAG1, ROS1, SS18, STAT6, TAF15, TCF12, TFE3, TFG, USP6, YWHAE.

RNA quality was not sufficient for NGS testing with the Archer FusionPlex Sarcoma genes by IonTorrent S5 Prime, an assay that is available for fusion gene detection.

The number of reads was not sufficient for an adequate test interpretation.

Considering the unusual behavior of this neoplasm and the uncertainty of its histology, we requested another revision of the tissue samples from a center with high expertise in sarcoma neoplasms. According to this last analysis, the thoracic mass was suggestive of a malignant, round-cell, high- grade mesenchymal neoplasm whose morphological and immunophenotypic characteristics were consistent with the diagnosis of ES. The sample’s cells were characterized by CD99 +; NKX2.2 +; cytokeratin AE1/AE3 -; desmin -; myogenin -; ETV4 -; S100 -.

The case was discussed in a national multidisciplinary tumor board specialized on sarcomas and, according to a sharing decision, the patient was candidate to an adjuvant chemotherapy.

The optimal therapeutic approach, suggested by the board, was an adjuvant chemotherapy with Vincristine, Doxorubicin and Cyclophosphamide (VDC) alternated to Ifosfamide and Etoposide (IE). Due to the fact that this intense regimen frequently leads to permanent infertility, a cryopreservation course was proposed to the young patient.

The treatment choice was communicated to the patient in order to share with her the benefit and the risks of the same. She expressed a strong childbearing desire so we also discussed the risks and benefits of postponing chemotherapy.

She decided to delay chemotherapy and the cryopreservation course in order to have a natural pregnancy.

Unfortunately, the patients relapsed after a few months. In fact, a CT scan performed during a close follow-up revealed a voluminous breast-like tissue of pathological significance in correspondence of the left lung apex with an inhomogeneous structure and areas of contextual necrosis of 68x50 mm in its maximum axial diameter. This tissue spread superiorly into the structures of the thoracic inlet contiguous to the first two costal arches and with the vascular structures of the left upper limb; posteriorly it invaded the costal pleura and medially infiltrated the smooth tissue of the anterior- superior mediastinum.

**Figure 4:**
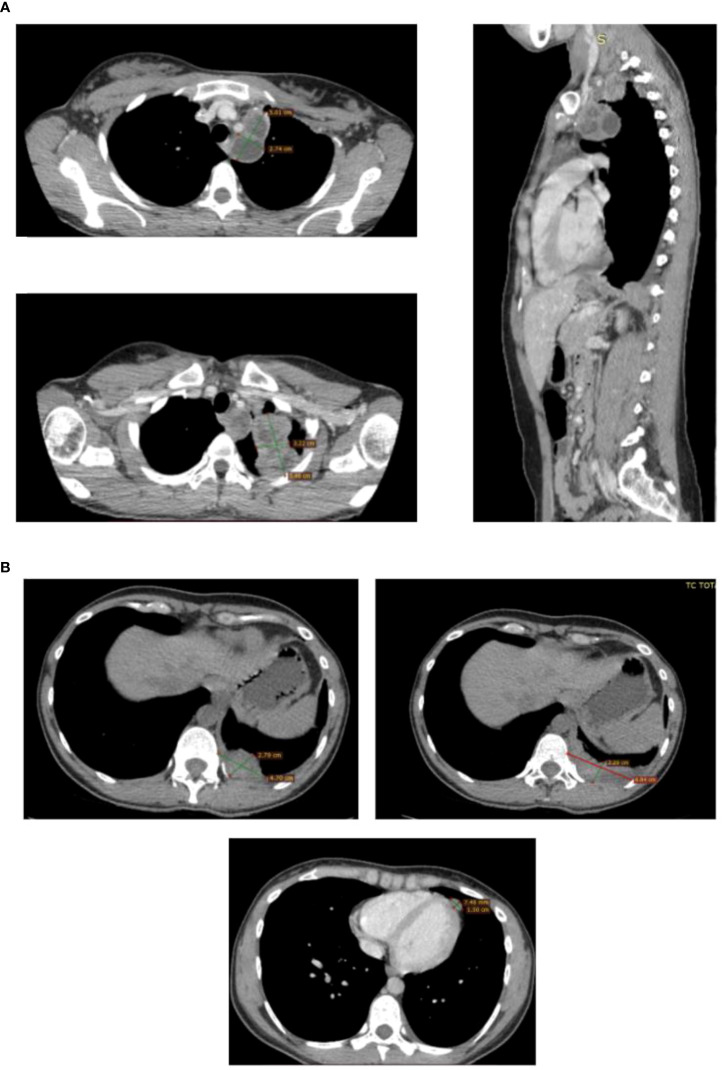
CT scans showing the presence and anatomical relation of the heteroplastic mass. CT scan detecting locoregional recurrence. **(A)** Necrotic pathological swelling in para-aortic region and breast-like pathological tissue in the left lung apex, axial and sagittal plane. **(B)** Solid nodulation along the pericardial sheet with reactive fluid flap and pathological mass along the left costo-vertebral pleura with pleural effusion flap.

There were confluent multiple pathological swellings with necrotic appearance extended caudally in the para-aortic region, whose largest diameter measured 51x30 mm. Solid hypervascular nodulations were found along the pericardial sheet, the largest nodulation measured 16x10 mm and was associated with reactive fluid flap. Pathological tissue of 47x23 mm was also detected along the left costo- vertebral pleura with contextual pleural effusion flap. All these findings were consistent with a locoregional disease recurrence (**Figure 4**).

The patient started the chemotherapeutic protocol VDC-IE. After four cycles of VDC-IE a radiological evaluation was performed with a CT scan. Fortunately, the voluminous solid breast-like tissue, previously described at the level of the left apex, the multiple pathological swellings of necrotic appearance, the solid nodulations along the pericardial leaflet and the pathological tissue along the left costo-vertebral pleura were no longer evident. This was considered a complete response and the patients continued her protocol with the last five cycles.

At the end of the ninth cycle the previously achieved complete response was maintained. The patient had experienced mild neutropenia and anemia, graded as G1, that did not lead to treatment discontinuation.

The case was discussed in the multidisciplinary board once again to evaluate the most suitable options to minimize the risk of a second recurrence and to strengthen the achieved optimal response. Among the feasible strategies there were: local radiation therapy, consolidation chemotherapy or close follow-up.

Considering the complete response previously achieved with a chemotherapy-based approach, after relapsing to up-front surgery, we proposed her high dose chemotherapy with subsequent autologous transplant (HDCT/ASCT) as consolidation treatment. The patient agreed and underwent two courses of mobilitiation chemotherapy followed by stem cell collection.

Notably, during the mobilization phase of the HDCT/ASCT, the patient experienced mild anemia, graded as G1, and moderate neutropenia, graded as G2 that did not required any medical interventions and did not lead to treatment interruption or discontinuation.

Overall, the treatment was well tolerated.

Finally, she received high dose chemotherapy with busulfan and melphalan and autologous transplantation.

To date, the patient is free from recurrences.

Remarkedly, the patient experienced an emotional journey through surgery chemotherapy and stem cell transplantation. Particularly, since the beginning she expressed a strong childbearing desire, unfortunately delayed by the required therapeutic interventions. Initially, the patient was prone to postpone chemotherapy adjuvant treatment to carry out a natural pregnancy. Subsequently, her perspective changed since the disease recurred and the fear of the unpredictable future forced her to a present-moment awareness. Of note, during the therapeutic iter, we offered the patient the possibility of cryopreservation to protect her desire. Nowadays, the patient is reshaping her future with more self-consciousness aiming to carry out a pregnancy and to find a renewed balance.

## Discussion

Ewing sarcoma is the second most common bone tumor among children and young adults. ES arises from bones but also from extraskeletal locations, even if more rarely.

ES patients should be addressed to referred high volume centers with recognised expertise in the diagnosis and treatment of rare entities.

The optimal management of ES requires ultraspecialized knowledge and resources.

EUropean Reference Network on Rare Adult Cancer (EURACAN) represents one of the most experienced and powerful reality connecting health care centers specialized in rare cancers.

The diagnosis and the management of extraskeletal ES follow the same principles as for bone ES (8). Nowadays, the diagnosis of ES is made by histological features and immunohistochemical markers positivity, even if their accuracy is low. Cluster of differentiation 99 (CD99) and Friend leukemia integration 1 (FLI-1) are currently accepted for the diagnosis of Ewing Sarcoma but they can also be expressed in a wide range of cancer entities different from ES.

FLI-1 is a transcription factor with a specificity and sensitivities, as a diagnostic marker for ES, that can vary from 63% to 100% and from 60% to 97%, respectively (9).

CD99 is a transmembrane molecule encoded by the pseudoautosomal gene MIC2. CD99 has been reported to have a marked effect on the migration, invasion and metastasis of tumor cells (10).

The definitive diagnosis is made on biopsy.

Molecularly, it is characterized by the presence of the translocation t (11,22) (q24;q12) that involves one of the members of the FET family (FUS/EWS/TLS), mostly EWSR1 and a member of the ETS (E26 transformation-specific or E-twenty-six) gene family, FLI1 in most cases (11).

This translocation is reciprocal and involves the Ewing sarcoma breakpoint region 1 (EWSR1) gene and almost always the FLI1 gene. EWSR1 and FLI1 merge in order to create a fusion gene that codifies for a fusion protein. The most frequent rearrangement is t (11,22) (q24;q12) that accounts for about 80% of ES while another 10% is represented by the fusion of EWSR1 with ETS-related gene (ERG) in the translocation t (21,22)(q11;q12) (12).

This molecular finding is mandatory in order to differentiate ES from other RCS.

Considering the rare incidence of these tumors, the histological assessment should be performed by specialized pathologists and a second revision should be encouraged to confirm the diagnosis.

Staging can be performed with both PET/CT scans and whole body magnetic resonance imaging (WB-MRI).

The treatment of ES involves combined modality therapy with chemotherapy and local therapy, both surgery and radiotherapy.

Surgery plays an important role in the management of ES and its aim is to ensure that the entire volume of tissue involved at diagnosis, and not only the tissue that remains after induction chemotherapy, is treated to guarantee an optimal local control.

Surgery is considered the best up-front modality also because local recurrence is frequent in ES, especially if radiotherapy (RT) is performed alone.

Generally, an induction chemotherapy is performed after the biopsy and prior to the local control with surgery and radiotherapy. The most used drugs are: vincristine (V), doxorubicin (D), cyclophosphamide (C)/ifosfamide (I) and etoposide (E). They all have proven activity in ES in large collaborative trials.

RT can be performed as up-front treatment when surgery can not guarantee a complete and radical excision or as adjuvant/neoadjuvant treatment (12).

As ES eventually relapses, first line therapy includes combined chemotherapy with vincristine, adriamycin, cyclophosphamide, ifosfamide and etoposide (13).

When a response, either partial or complete, to this salvage therapy is gained a consolidation therapy with high dose chemotherapy followed by autologous transplantation can be taken into consideration (14).

This regimen is characterized by different phases: stem cells stimulation, stem cells collection, called apheresis, stem cell preservation, high dose chemotherapy, stem cells transplantation and engraftment.

In the stem cell stimulation phase, also known as mobilization, three to four cycles of chemotherapy regimen with cyclophosphamide and etoposide followed by daily injections of Granulocytes-colony stimulating factor (G-CSF) are administered. Subsequently, Peripheral blood stem cells (PBSC) are collected by continuous apheresis.once the peripheral blood CD34+ count is at least 5 cells/l.

The stem cells are frozen using liquid nitrogen in a process known as cryopreservation.

Morever, autologous transplantation is started with high dose chemotherapy with busulfan and melphalan and peripheral blood stem cells are infused on day 0.

Supportive care included: antibiotic, antiviral and antimycotic drugs are used in order to reduce the risk of opportunistic and not opportunistic infections.

Furthermore, a low-dose heparin prophylaxis given by continuous infusion at 100 U/kg can be considered to prevent a fearful consequence of HDCT/ASCT as sinusoidal obstruction

Due to the high rates of relapse of ES, there is an unmet need in finding therapeutic opportunities for patients whose disease recur after standard first line therapies. Besides, the phase III rEECur trial provided the first randomized evidence of activity between regimens in an extremely rare disease as ES. Indeed, the four most common regimen used in recurrent and primary refractory ES, irinotecan plus temozolomide (IT), gemcitabine plus docetaxel (GD), high dose ifosfamide (hd-IFO) and topotecan plus cyclophosphamide (TC) were compared one with the other.

The trial was designed to discontinue the least successful treatment arms after 50 and then 75 patients had been randomly assigned and their results evaluated, respectively, regardless of the treatment difference magnitude.

At the first and second interim analysis the two arms, GD and IT, were discontinued.

Between hd-IFO and TC, the first showed better progression free survival and overall survival in the last analysis.

Currently, the rEECur trial is recruiting patients to hd-IFO and another, recently added arm, carboplatin plus etoposide (CE). Data is still awaited, as a molecularly targeted agent arm is planned (15). Moreover, as intensive chemotherapy regimens represent the essential backbone of ES treatment, the toxicity as well as the quality of life evaluation has to be considered. In particular, whereas our patient experienced mild neutropenia, and anemia that did not lead to treatment discontinuation, commonly the chemotherapeutic protocol adopted has been associated with high toxicity rate (16). Besides, the rEECur trial showed different toxicity profiles between regimens as hd-IFO arm compared with TC arm was more likely associated with 3/4 encephalopathy and kidney impairment and patients were more likely to experience treatment discontinuation. Therefore, a multidisciplinary approach is required to better select patients according to performance status, organ functions, time relapsed since prior therapy, and to manage the related adverse events that could eventually affect treatment efficacy.

## Conclusion

Although ES arising from mediastinum is extremely rare, it should be considered when a differential diagnosis of a mediastinal mass has to be made in adult patients who may benefit from an aggressive multimodality treatment.

As far as we know, only a few cases of primary mediastinal extraskeletal Ewing sarcoma have been reported and currently there is no consensus on the optimal treatment strategy to adopt. The main issue is represented by the lack of concordance in the histological diagnosis which eventually leads to a delayed diagnosis. Moreover, the sequential combination of chemotherapeutic protocols required, as well as the toxicities related to management, needs a long-established expertise and a multidisciplinary approach in order to provide the patient with the most appropriate treatment selection.

## Data availability statement

The original contributions presented in the study are included in the article/supplementary material, further inquiries can be directed to the corresponding author/s.

## Ethics statement

The authors are accountable for all aspects of the work in ensuring that questions related to the accuracy or integrity of any part of the work are appropriately investigated and resolved. The patient signed informed consent for the use of all the reported data. The anonymity was assured. Written informed consent was obtained from the patient for publication of this case report and accompanying images. A copy of the written consent is available for review by the editorial office of this journal. The authors are accountable for all aspects of the work in ensuring that questions related to the accuracy or integrity of any part of the work are appropriately investigated and resolved.

## Author contributions

AC and RB contribute to the writing of the case report. MO, GP, SDP, and MG contribute to the idea of the case report and the supervision of the manuscript. MT, FS, AP, and FP, resources and data curation. RM, EP, and PDP revision of the manuscript and editing. MM contributes to the anatomopathological support in the writing of the case report and supply all the figures regarding this aspect. All authors contributed to the article and approved the submitted version.
